# Prompting Fab Yeast Surface Display Efficiency by ER Retention and Molecular Chaperon Co-expression

**DOI:** 10.3389/fbioe.2019.00362

**Published:** 2019-11-26

**Authors:** Meng Mei, Junhong Li, Shengchen Wang, Ki Baek Lee, Brent L. Iverson, Guimin Zhang, Xin Ge, Li Yi

**Affiliations:** ^1^State Key Laboratory of Biocatalysis and Enzyme Engineering, Hubei Collaborative Innovation Center for Green Transformation of Bio-Resources, Hubei Key Laboratory of Industrial Biotechnology, School of Life Sciences, Hubei University, Wuhan, China; ^2^Department of Chemical and Environmental Engineering, University of California, Riverside, Riverside, CA, United States; ^3^Department of Chemistry, University of Texas, Austin, TX, United States

**Keywords:** yeast surface display, Fab, divergent promotor, ER retention sequence, molecular chaperone

## Abstract

For antibody discovery and engineering, yeast surface display (YSD) of antigen-binding fragments (Fabs) and coupled fluorescence activated cell sorting (FACS) provide intact paratopic conformations and quantitative analysis at the monoclonal level, and thus holding great promises for numerous applications. Using anti-TNFα mAbs Infliximab, Adalimumab, and its variants as model Fabs, this study systematically characterized complementary approaches for the optimization of Fab YSD. Results suggested that by using divergent promoter *GAL1-GAL10* and endoplasmic reticulum (ER) signal peptides for co-expression of light chain and heavy chain-Aga2 fusion, assembled Fabs were functionally displayed on yeast cell surface with sigmoidal binding responses toward TNFα. Co-expression of a Hsp70 family molecular chaperone Kar2p and/or protein-disulfide isomerase (Pdi1p) significantly improved efficiency of functional display (defined as the ratio of cells displaying functional Fab over cells displaying assembled Fab). Moreover, fusing ER retention sequences (ERSs) with light chain also enhanced Fab display quality at the expense of display quantity, and the degree of improvements was correlated with the strength of ERSs and was more significant for Infliximab than Adalimumab. The feasibility of affinity maturation was further demonstrated by isolating a high affinity Fab clone from 1:10^3^ or 1:10^5^ spiked libraries.

## Introduction

Monoclonal antibodies (mAbs) represent the fastest growing class of therapeutics in the last decades. By the end of 2018, at least 116 mAb-based biopharmaceutical products are active on the market (Walsh, 2018; DeFrancesco, 2019). Notably, mAbs occupies seven spots out of the top ten best-selling drugs in 2018 (Urquhart, 2019). As 54 new mAbs in late-stage clinical trials are under regulatory review, it is expected that mAb-based products will continue to dominate the biopharmaceutical approvals in the near future. From the biotechnology viewpoint, an essential development step is the affinity maturation of lead mAbs to achieve high potencies desirable for therapeutic practices. Following the generation of combinatorial libraries, a high-throughput selection/screening method needs to be exploited to isolate mAb variants with improved affinities. Compared to selection approaches, which rely on overall binding strength such as phage panning, fluorescence activated cell sorting (FACS) is advantageous by providing quantitative analysis of each library member. During subsequent rounds of sorting, the concentration of a fluorophore-labeled antigen can be fine-tuned in a real-time manner, leading to efficiently distinguish high affinity clones from others. In addition, the multiparameter nature of FACS allows to normalize the difference on antibody expression levels among cells and/or various antibody clones. Accordingly, a dual color sorting with one channel for antibody expression and the other for antigen binding has been proven highly effective for enriching affinity improved clones (Feldhaus et al., 2003; van den Beucken et al., 2003).

For FACS, recombinant antibodies or their fragments must be present on cell surface. Compared to mammalian cells, yeast has been widely used for antibody surface display due to its low cost, ease to handle, and facile construction of antibody libraries (Boder and Wittrup, 1997; Pepper et al., 2008). Derived from human immunoglobulin G (IgG), the design of single-chain variable fragment (scFv) links a heavy chain variable domain (V_H_) with its associated light chain variable domain (V_L_) via a flexible linker. As the smallest human antibody fragment with binding function, scFv can be efficiently displayed on yeast cell surface such as by fusion with a-agglutinin aga2 (Boder and Wittrup, 1997). Consequently, yeast surface display (YSD) of scFv achieved great successes for antibody discovery and engineering using either immunized or naïve/synthetic libraries (Feldhaus et al., 2003; Miller et al., 2008). However, the conformation of V_H_ and V_L_ domains in scFv format may not be the same as in its natural IgG, in which the heavy and light chains also interact through their constant heavy 1 (C_H_1) and constant light (C_L_) domains. Although such conformational variations are usually subtle, its impact on binding affinity can be substantial and problematic for affinity maturation studies (Casadevall and Janda, 2012). In fact, it is not uncommon that significant potency loss happens when an affinity matured scFv clone is converted back to its associated IgG (Steinwand et al., 2014; Yang et al., 2018). As the antigen-binding fragment (Fab) contains half heavy chain (V_H_-C_H_1) and entire light chain (V_L_-C_L_), this format can reserve V_H_ and V_L_ domains in their intact conformations. Therefore, it is argued that the best combination for affinity maturation is to display Fabs on yeast surface and to screen by FACS.

Since the initial studies of YSD in 1990s (Boder and Wittrup, 1997), recent researches have demonstrated the feasibility of displaying Fabs (Rosowski et al., 2018; Wang et al., 2018), and full-length IgGs (Rhiel et al., 2014) on yeast cell surface. These developments apply different technologies including bi-directional promoter design for co-expression (Rosowski et al., 2018), type II restriction enzymes for library construction (Roth et al., 2019), immobilized ZZ domain for surface display (Rhiel et al., 2014), and leucine-zipper interactions for Fab assembly (Wang et al., 2018). It has been proven that Fab was more reliable than scFv for YSD (Sivelle et al., 2018), and Fab YSD was suitable for antibody affinity maturation (Yang et al., 2018). Despite these tremendous advances, systematic study for Fab YSD optimization is still lacking.

One characteristic of protein production in yeast cells is that various molecular chaperons exist in the endoplasmic reticulum (ER) to facilitate the protein folding and post-translational modifications. Kar2p, also known as BiP, is a major member of the Hsp70 chaperone family, which binds to unfolded polypeptide chains and mediates protein folding within the ER (Rose et al., 1989; Valkonen et al., 2003; Hernandez-Elvira and Torres-Quiroz, 2018). Only correctly folded proteins can be released from Kar2p, while abnormally folded or improperly assembled proteins are retained by Kar2p for later degradation. In addition, ER-associated protein disulfide isomerase (Pdi1p) catalyzed the disulfide bonds formation in eukaryotic cells (Farquhar et al., 1991; Niu et al., 2016; Beal et al., 2019). These molecular chaperons are crucial for the Fab assembly, whose efficiency depends on the correct folding of V_H_-C_H_1 and V_L_-C_L_ domains as well as the formation of intra- and inter-molecular disulfide bonds. Moreover, ER retention, mediated by characteristic ER retention sequence (ERS), is a mechanism that ensures only properly folded/assembled proteins are exported from the ER to the Golgi (Munro and Pelham, 1987). This phenomenon provides a means of quality control during protein synthesis, maturation and assembly, because misfolded or incorrectly assembled proteins are retained in the ER and targeted for degradation (Ellgaard et al., 1999). Our previous work on characterization of HDEL-type ERS suggested that the ERS sequence HDEL exhibited a protein ER retention ability 2-fold stronger than that of KDEL but was 50% of FEHDEL, the strongest endogenous ERS in *Saccharomyces cerevisiae* (Munro and Pelham, 1987; Mei et al., 2017). Further systematic study of all endogenous ERS indicated that an engineered sequence WEHDEL could confer 2-fold stronger retention ability than FEHDEL. Building on above knowledge, using anti-tumor necrosis factor α (TNFα) mAbs Infliximab (Keane et al., 2001), Adalimumab (D2E7), and its variants (Rajpal et al., 2005) as model Fabs, this study characterized the impacts of molecular chaperons Kar2p and Pdi1p and four ERSs, i.e., WEHDEL, FEHDEL, HDEL, and KDEL, on Fab YSD efficiency. In addition, the feasibility of Fab maturation through high-throughput screening was demonstrated by FACS sorting of large spiked libraries.

## Materials and Methods

### Construction of Fab and scFv Yeast Surface Display Vectors

Genes encoding V_H_-C_H_1 and V_L_-C_L_ fragments (Salfeld et al., 2003; Rajpal et al., 2005) were chemically synthesized, amplified by PCR and cloned to *Pst*I/*Eco*RI sites and *Bam*HI/*Xho*I sites, respectively, on pESD (Yi et al., 2015) to generate pESD-Fab. pESD-HC (heavy chain only) and pESD-LC (light chain only) were also generated as controls. Fragments encoding Adalimumab variant scFvs, V_L_-(G_4_S)_3_-V_H_, were assembled by overlapping PCR and cloned to *Pst*I/*Eco*RI sites on pESD to generate pESD-scFv. Kar2p and Pdi1p wild-type (wt) genes were amplified by PCR using the genome of *S. cerevisiae* EBY100 as the templates and fused with protomer *GAL1* region by overlapping PCR. Using Kar2p and Pdi1p wt genes as the templates, their mutants were generated through site mutagenesis. Obtained *GAL1*-Kar2p/Pdi1p cassettes were cloned to *Kpn*I/*Bgl*II sites on pESD-Fab to give pESD-Fab-Kar2p/Pdi1p. Additional *GAL1*-Kar2p cassette was cloned to *Sac*I site on pESD-Fab-Pdi1p to give pESD-Fab-Pdi1p-Kar2p. ERSs were introduced at C-termini of light chains by extension PCR and cloned to pESD-Fab to give pESD-Fab-ERS. All cloning works were performed in *Escherichia coli* XL-Gold and confirmed by DNA sequencing.

### Expression and Purification of TNFα-His_6_

Gene of human TNFα (NCBI ID: 7124) was chemically synthesized, cloned to pET-28a, and transformed to *E. coli* BL21 (DE3) cells for culture in LB medium at 37°C. When OD_600_ reached 0.6–0.8, cells were induced with 0.5 mM IPTG at 18°C for 20 h. After induction, cells were collected and resuspended in lysis buffer containing 50 mM Tris (pH 8.0), 150 mM NaCl, 0.5 mg/mL lysozyme, 1 mM PMSF, and 10 mM imidazole for 1 h on ice, followed by sonication. Cell debris was then removed by centrifugation at 10,000 ×g at 4°C for 20 min, and the supernatant containing TNFα-His_6_ was subjected to Ni-NTA affinity purification at 4°C (Qiagen, Valencia, CA, USA). Purity of produced TNFα-His_6_ was tested by SDS-PAGE, and its concentration was measured by UV absorbance.

### Flow Cytometry Analysis of Displayed Fabs and scFvs on Yeast Cell Surface

Generated yeast surface display plasmids were transformed to *S. cerevisiae* EBY100 competent cells (Cooper and Hausman, 2000). Transformed cells were cultivated in SD-CAA medium (20 g/L D-glucose, 6.7 g/L yeast nitrogen base, 5 g/L casamino acids, 5.4 g/L Na_2_HPO_4_, 8.6 g/L NaH_2_PO_4_·H_2_O, pH 7.4) at 30°C with shaking at 230 rpm. When OD_600_ reached 0.5–1.0, cells were collected by centrifugation at 3,000 ×g for 2 min and inoculated to SG-CAA medium (20 g/L galactose, 6.7 g/L yeast nitrogen base, 5 g/L casamino acids, 5.4 g/L Na_2_HPO_4_, 8.6 g/L NaH_2_PO_4_·H_2_O, pH 7.4). After induction at 18°C for 48 h, cells were harvested by centrifugation, washed three times with PBS (pH 7.4), supplemented with 0.5% BSA and 1 mM EDTA, and re-suspended as 0.1 OD_600_ cells per 20 μL PBS (pH 7.4), 0.5% BSA. For Fab and scFv display analysis, cells were incubated with 0.1 μM anti-HA-FITC and/or 0.1 μM anti-FLAG-iFlor647 (GenScript, Nanjing, China) for 15 min in dark. Flow cytometry analysis was performed by using Beckman Coulter CytoFLEX (Brea, CA) equipped with 488 and 633 nm lasers and 525/40 and 660/20 nm band-pass filters. To test binding function of cell surface displayed antibody fragments, cells were incubated with 1 pM −20 nM purified TNFα-His_6_ at 25°C for 30 min and subsequently labeled with 0.1 μM anti-His_6_-iFluor647 (GenScript, Nanjing, China). Percentages of TNFα^+^ cells were quantified by FACS. The cells carrying Fab heavy chain (V_H_-C_H_1) without light chain and light chain (V_L_-C_L_) without heavy chain were used as controls.

### FACS Enrichment for High Affinity Fab Clone From Spiked Libraries

Cells bearing Fab cb2-6 and Fab D2E7 display vectors were mixed at 1:10^3^ or 1:10^5^ ratios. After cultivation and induction, mixed cells were labeled with TNFα-His_6_ of predetermined concentrations, and subsequently labeled with 0.1 μM anti-His_6_-iFluor647 and 0.1 μM anti-HA-FITC. Sorting was performed in the single cell mode by using Beckman Coulter MoFlo XDP flow cytometer (Brea, CA, USA) equipped with 488 and 633 nm lasers and 525/40 and 660/20 nm band-pass filters. In each round, 10^7^-10^9^ cells were sorted, and 0.6–1.0% cells with the highest FITC/iFluor647 double signals were collected. Collected cells were cultivated in SD-CAA and induced in SG-CAA for the next round of sorting. Aliquots of collected cells were also recovered on SD-CAA plates for monoclonal analysis. Yeast plasmids were extracted by using Zymolyase (Amsbio, Abingdon, UK) and transformed into *E. coli* XL-Gold for amplification and sequence analysis.

### Yeast Total RNA Extract and qRT-PCR

After galactose induction, total RNA was extracted from yeast cell samples by using RNAiso kit (TaKaRa Bio, Kusatsu, Japan). Reverse transcription was performed by using PrimeScript II first-strand cDNA synthesis kit (TaKaRa Bio) with Random 6 mers. The cDNA levels of *Kar2p* and *Pdi1p* were then analyzed with CFX real-time PCR (Bio-Rad, Hercules, CA, USA). Relative expression levels against endogenous *Taf10* were determined with efficiency correction and associated technical errors were calculated. The final results were normalized by the relative mRNA levels of *Kar2p* and *Pdi1p* in EBY100 cells.

## Results

### Design for Fab Yeast Surface Display

**Figure 1:**
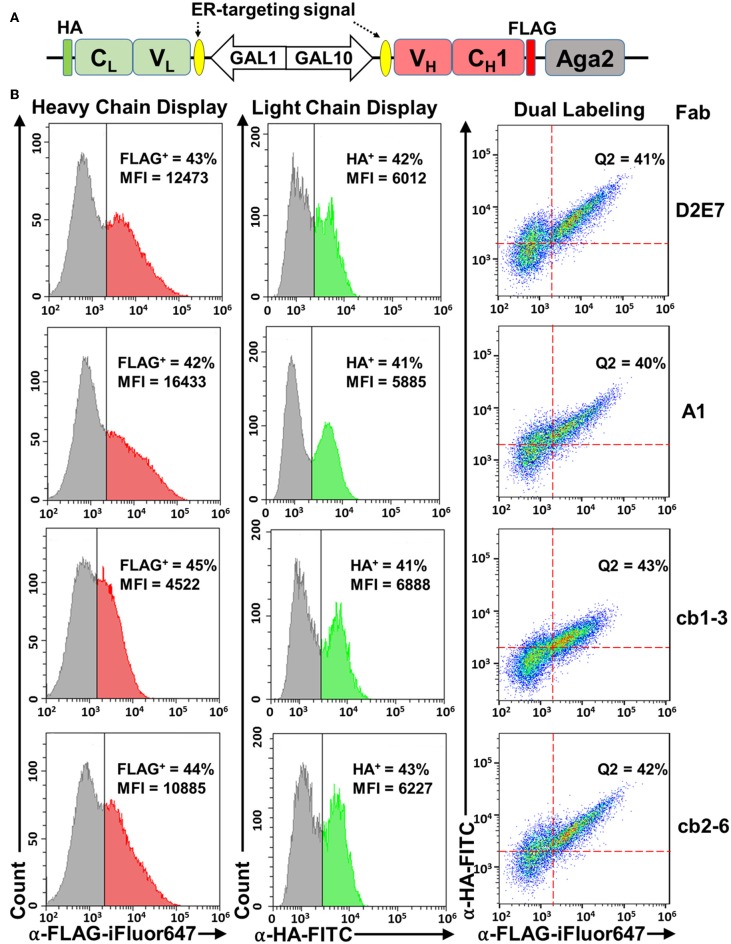
Fab yeast surface display using a divergent promoter, ER-targeting signals, and Aga2 fusion. **(A)** pESD-Fab. Expression cassettes for light (V_L_-C_L_) and heavy (V_H_-C_H_1) chains were located downstream of *GAL1-GAL10* promoter and endoplasmic reticulum (ER)-targeting signals. Light chain was fused with a HA tag, and heavy chain was fused with a FLAG tag to the N-terminus of Aga2. **(B)** Validation of heavy chain display (left panels), light chain display (middle panels), and Fab assembly (right panels) on yeast cell surface by flow cytometry. Expression was induced by 20 g/L galactose and cells were incubated with 0.1 μM anti-FLAG-iFluor647 and/or 0.1 μM anti-HA-FITC antibodies. Fab display of Adalimumab (D2E7) and three variants were tested. Percentages of cells displaying assembled Fabs were shown as Q2 (FLAG^+^/HA^+^ double positive). MFI, mean fluorescence intensity.

For successful display of a Fab, its heavy chain (V_H_-C_H_1) and light chain (V_L_-C_L_) need to be co-expressed. In our design, a divergent *GAL1-GAL10* promoter derived from previous studies (West et al., 1987; Boder et al., 2005; Jiang and Boder, 2010) is exploited by cloning Fab heavy chain and light chain at downstream of *GAL10* and *GAL1*, respectively (Figure 1A). Therefore, in the presence of galactose, expression of both chains will be induced simultaneously. Yeast endoplasmic reticulum (ER) signal sequences are included at N-termini of both chains for their translocation and secretory expression. Once translated, Fab assembly between heavy and light chains, especially via the intermolecular disulfide connecting C-termini of C_H_1 and C_L_, is critical for its binding function (Padlan et al., 1986). Since protein disulfide-bond formation in eukaryotic cells mainly occurs in ER (Frand et al., 2000), targeting ER via signal sequences also enhances Fab assembly. For surface display, V_H_-C_H_1 is fused to the N-terminus of Aga2p, which leads transportation to cell surface through the a-agglutinin system by a disulfide linkage to the cell wall-anchored Aga1p (Boder and Wittrup, 1997). In addition, a FLAG tag and a HA tag are introduced to the heavy and light chain expression cassettes, respectively, for facile detections (Figure 1A).

### Display of Assembled Fabs on Yeast Cell Surface

Fab display plasmids of anti-TNFα mAb D2E7 and its variants were constructed and transformed into *S. cerevisiae* EBY100 cells. After cultivation and induction with 20 g/L galactose, cells were analyzed by flow cytometry. To detect surface displayed D2E7 heavy chain, which was fused with a FLAG tag to the N-terminus of Aga2, cells were incubated with 0.1 μM iFluor 647 conjugated anti-FLAG antibodies. FACS results showed the successful anchoring of D2E7 heavy chain on yeast cell surface, with 43% cells were FLAG^+^ (Figure 1B, left panel). In contract, <0.6% cells bearing D2E7 light chain without its heavy chain gene were FLAG^+^ Figure S1A). Similarly, the display of HA-tagged D2E7 light chain was detected with 0.1 μM FITC conjugated anti-HA antibodies, and the results indicated that 42% cells were HA^+^ (Figure 1B, middle panel), while <0.4% cells bearing D2E7 heavy chain without its light chain gene were HA^+^ (Figure S1B). In addition, <0.6% cells bearing D2E7 light chain without its heavy chain gene were HA^+^ (Figure S1C). As D2E7 light chain expression cassette (V_L_-C_L_-HA) did not possess the fusion partner Aga2 for anchoring, display of D2E7 light chain on cell surface was presumably caused by its Fab assembly between secreted light chain and anchored heavy chain. To confirm the presence of both chains on individual cells, induced cells were further dual labeled with 0.1 μM anti-FLAG-iFluor647 and 0.1 μM anti-HA-FITC. FACS results revealed that 41 ± 3% cells were FLAG^+^/HA^+^ double positive (Figure 1B, right panel), suggesting the display of intermolecularly assembled D2E7 Fab. Display profiles of D2E7 variants A1, cb1-3, and cb2-6 were also characterized in the same approaches. Results suggested the similar display levels for their heavy chains (42–45% FLAG^+^, Figure 1B, left panel), their light chains (41–43% HA^+^, Figure 1B, middle panel), and assembled Fabs (40–42% FLAG^+^/HA^+^ double positive, Figure 1B, right panel). Notably, the mean fluorescence intensity (MFI) of FLAG^+^ cells for cb1-3 was considerably less than other tested Fab clones, suggesting that cb1-3 presumably exhibited a lower expression level or less display efficiency of its heavy chain.

### Yeast Surface Displayed Fabs Were Functional

**Figure 2:**
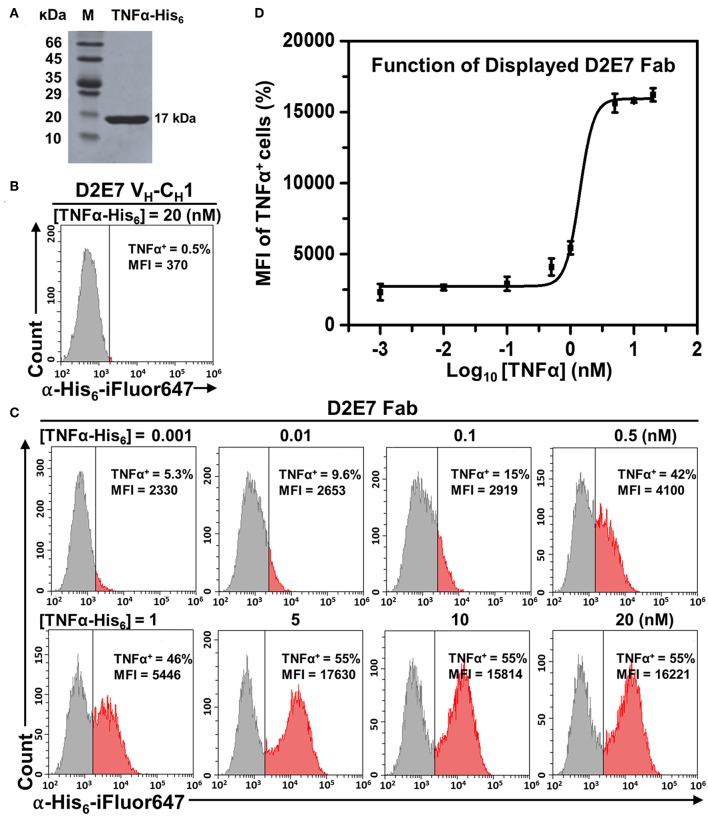
Function characterizations of displayed Fab D2E7. **(A)** Recombinant production of human TNFα as a His_6_ tagged protein. **(B)** Flow cytometry analysis of cells carrying the gene of Adalimumab (D2E7) heavy chain (V_H_-C_H_1) without its light chain. Expression was induced by 20 g/L galactose and cells were labeled with 20 nM TNFα-His_6_ and 0.1 μM anti-His_6_-iFluor647. **(C)** Flow cytometry analysis of cells carrying D2E7 Fab display construct. Induced cells were labeled with 1 pM −20 nM TNFα-His_6_ and 0.1 μM anti-His_6_-iFluor647 for TNFα^+^ cell quantification. **(D)** Sigmoidal curves depicting fluorescence intensities of TNFα^+^ cells as a function of TNFα concentrations. Data are presented as mean ± S.E. (*n* = 3 independent experiments). MFI, mean fluorescence intensity.

To test binding functions of displayed D2E7 Fab and its variants, human TNFα as a His_6_ tagged protein was recombinantly produced in *E. coli* with a typical yield of 24 mg purified TNFα-His_6_ per liter of culture (Figure 2A). Cells bearing D2E7 Fab construct were incubated with 1 pM−20 nM TNFα-His_6_ followed by labeling with 0.1 μM iFluor 647 conjugated anti-His_6_ antibodies. In parallel, cells displaying D2E7 heavy chain without its light chain was prepared as the control. FACS results indicated that, when 20 nM TNFα-His_6_ was used, only 0.5% of cells displaying D2E7 V_H_-C_H_1 were TNFα^+^ (Figure 2B), suggesting that TNFα cannot be recognized by either unassembled D2E7 heavy chain or yeast host cell EBY100. In contrast, when D2E7 Fab was displayed, after incubation with even 1 pM TNFα-His_6_, 5.3% cells were TNFα^+^ (Figure 2C). The percentages of TNFα^+^ cells increased with higher TNFα-His_6_ concentrations: 9.6% at 10 pM, 15% at 100 pM, 42% at 500 pM, and 46% at 1 μM. The proportion of TNFα^+^ cells became plateaued 55% when 5–20 nM TNFα-His_6_ was applied, suggesting that under used culture and induction conditions, 55% cells displayed functional D2E7 Fab. Plotting MFIs of TNFα^+^ cells over TNFα concentrations demonstrated a sigmoidal correlation (Figure 2D). Dose-response relationships on TNFα^+^ cell percentages and associated MFIs were also measured for D2E7 variants A1, cb1-3, and cb2-6. Results showed that ~60% cells displayed functional Fabs (Figure S2A) with expected sigmoidal curves between MFIs and TNFα concentrations (Figures S2B–D). All these results suggested that yeast surface displayed Fabs exhibited their specific binding functions. Notably, when 1–100 pM TNFα was used for cell labeling, MFIs of TNFα^+^ cells were higher for variants cb1-3 and cb2-6 than D2E7 (Figure 2C, Figure S2A), likely due to their improved affinities (Rajpal et al., 2005).

### Functional Display Efficiency Was Improved by Co-expression of Molecular Chaperones

**Figure 3:**
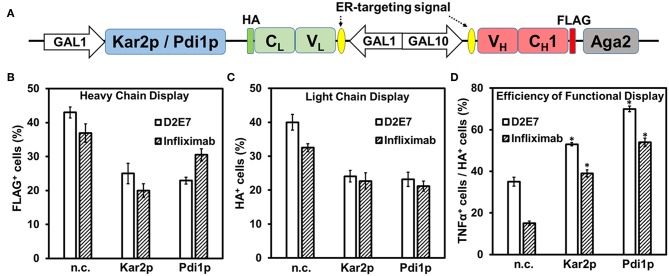
Effects of molecular chaperones on Fab functional display. **(A)** Co-expression of Kar2p or Pdi1p was under the control of an additional *GAL1* promoter on pESD-Fab-Kar2p/Pdi1p. 0.1 μM anti-FLAG-iFluor647, 0.1 μM anti-HA-FITC, and 0.1 nM TNFα/0.1 μM anti-His_6_-iFluor647 were used for labeling induced cells. Display of heavy chain (FLAG^+^, **B**), display of light chain (HA^+^, **C**), efficiencies of functional display (defined as the percentage ratios between TNFα^+^ cells and HA^+^ cells, **D**) were presented as mean ± S.E. (*n* = 3 independent experiments) with Student's *t*-test being performed, *P < 0.05.

As molecular chaperones in the ER assist folding of newly synthesized proteins and prevent them from misfolding and/or formation of aggregates (Hartl and Hayer-Hartl, 2002), we next investigated the effects of a Hsp70 family member Kar2p (Rose et al., 1989) on Fab surface display. To achieve co-expression of Kar2p, its gene was cloned downstream of an additional *GAL1* promoter allowing its simultaneous induction with galactose (Figure 3A). When EBY100 cells producing D2E7 Fab and Kar2p were labeled with anti-FLAG-iFluor647 or anti-HA-FITC for detecting displayed heavy chain or light chain, results revealed that co-expression of Kar2p in fact reduced the percentages of cells displaying D2E7 heavy chain from 43 ± 2% to 25 ± 3% and cells displaying assembled D2E7 Fab from 40 ± 2% to 24 ± 2% (Figures 3B,C, Table S1). MFIs of FLAG^+^ cells and HA^+^ cells also decreased when Kar2p was co-expressed, suggesting that less amounts of heavy chain and assembled Fab were displayed. To test the binding function of displayed D2E7 Fab, induced cells were sequentially labeled with 0.1 nM TNFα-His_6_ and 0.1 μM anti-His_6_-iFluor647. We used the ratio of TNFα^+^ percentage (cells displaying functional Fab) over HA^+^ percentage (cells display assembled Fab) to assess the efficiency of functional display. As results shown in Figure 3D, co-expression of Kar2p increased the efficiency of functional display of D2E7 Fab from 36 ± 2% to 53 ± 1%.

To further evaluate the contribution of Kar2p on Fab folding, we constructed inactive Kar2p mutants as controls. As a Hsp70 chaperon, Kar2p's nucleotide binding domain carries ATPase activity to promote the interaction between its substrate binding domain and unfolded protein substrates. Therefore, by alanine substitutions of its ADP/ATP recognizing residues E313/K316/S320 (Yan et al., 2011), or incorporating the mutants of temperature-sensitive alleles C63Y/F196L/G417S (Kimata et al., 2003), inactive Kar2p_E313A/K316A/S320A_ and Kar2p_C63Y/F196L/G417S_ were generated. Same strategy was used that co-expression of these Kar2p variants was under the control of additional *GAL1* promoter (Figure S3A). Co-expression of Kar2p variants with D2E7 Fab resulted in drops of cell percentages displaying D2E7 heavy chain (to 20 ± 2% and 23 ± 2% respectively) and assembled D2E7 Fab (to 19 ± 2% and 21 ± 3%) (Figures S3B–D, Table S1). As expected, these biochemically inactive Kar2p variants failed to improve display efficiencies of functional D2E7 Fab, as TNFα^+^ %/HA^+^ % ratios of 32 ± 4% and 38 ± 2% were statistically indifferent from the D2E7 Fab producing cells without chaperon co-expression.

Often as the rate-limiting step of protein folding in ER, disulfide bond formation is critical for V_H_ and V_L_ domain folding and their assembly to form a functional Fab. Therefore, we further tested the effects of ER-associated protein-disulfide isomerase (Pdi1p) on Fab surface display. Similar to Kar2p, Pdi1p gene was cloned downstream of a *GAL1* promoter for its co-expression. Induced cells were labeled with anti-FLAG-iFluor647 for detecting heavy chain display, anti-HA-FITC for detecting Fab assembly, and TNFα-His_6_ and anti-His_6_-iFluor647 for detecting Fab function. Results indicated that when Pdi1p was co-expressed, the cell percentage displaying D2E7 heavy chain decreased from 43 ± 2% to 23 ± 1%, and the cell percentage displaying D2E7 Fab decreased from 40 ± 2% to 23 ± 2%. However, the functional display efficiency (defined as the ratio of TNFα^+^ cells over HA^+^ cells) enhanced from 36 ± 2% to 70 ± 1% (Figure 3). We also constructed Pdi1p_C4S_ mutant by changing the four catalytic cysteines of its thioredoxin-like domains to serines (Tian et al., 2006; Wang et al., 2013), and Pdi1p_C6S_ mutant which carried two additional Cys->Ser substitutions important for Pdi1p re-oxidation mediated by sulfhydryl oxidase ER oxidoreductin 1 (Ero1p) (Frand and Kaiser, 2000; Niu et al., 2016). Co-expression of inactive Pdi1p_C4S_/Pdi1p_C6S_ with D2E7 Fab decreased cell percentage displaying its heavy chain and assembled Fab without improvements on functional display efficiencies (Figure S3, Table S1).

Infliximab is another therapeutic mAb targeting TNFα (Keane et al., 2001), however its display as a scFv on yeast cell surface has been proved difficult (Sivelle et al., 2018). To investigate whether co-expression of Kar2p or Pdi1p can improve the display quality of this challenging antibody clone, we constructed Infliximab Fab yeast display and its co-expression vectors for Kar2p or Pdi1p (Figure 3A). Similar to testing displayed D2E7 Fab, induced cells were labeled and measured for Infliximab heavy chain display (FLAG^+^ cells), Fab assembly (HA^+^ cells), and its function (TNFα^+^ cells). Results showed that when Kar2p or Pdi1p was co-expressed, while the percentages of cells displaying Infliximab heavy chain decreased from 37 ± 3% to 20 ± 2% or 30 ± 2%, and the percentages of cells displaying Infliximab Fab decreased from 33 ± 1% to 23 ± 2% or 21 ± 2%, its efficiency of functional display (TNFα^+^/HA^+^) increased from 15 ± 1% to 39 ± 2% or 53 ± 2%, representing an 2.6- or 3.6-fold improvement, respectively (Figures 3B–D, Table S1). Applying inactive Kar2p or Pdi1p variants toward Infliximab Fab failed to improve its functional display efficiencies (Figure S3, Table S1). Collectively, results with two tested anti-TNFα clones suggested that molecular chaperone Kar2p or Pdi1p significantly improved the quality of displayed Fabs.

We also tested the effects of dual chaperones Kar2p and Pdi1p co-expression on Fab YSD. Simultaneous co-expression of Kar2p and Pdi1p was under the control of additional *GAL1* promoters on pESD-Fab-Pdi1p-Kar2p. Results indicated that the efficiencies of functional display (TNFα^+^/HA^+^) were improved from 36 and 15% to 65 and 56% for D2E7 and Infliximab Fab, respectively, while their light chain display amounts (as HA^+^) decreased from 40 and 33% to 20 and 16% (Figure S4). These results suggested that, similar to the effects of Kar2p/Pdi1p alone, simultaneous co-expression of these two chaperones improved the quality of displayed Fabs at the expense of display quantity.

### Functional Display Efficiency Was Improved by Fusion With ER Retention Sequences

**Figure 4:**
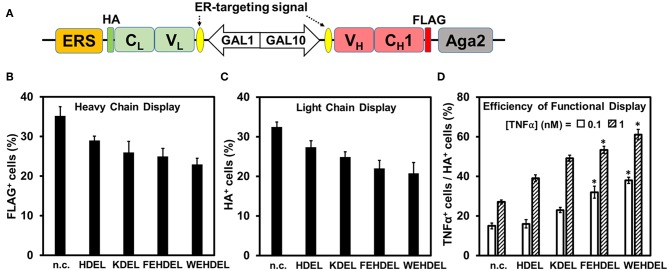
Effects of ER-retention sequence (ERS) on Infliximab Fab functional display. **(A)** ERSs were fused to the C-terminus of Infliximab light chain. 0.1 μM anti-FLAG-iFluor647, 0.1 μM anti-HA-FITC, and 0.1–1.0 nM TNFα/0.1 μM anti-His_6_-iFluor647 were used for labeling induced cells. Display of heavy chain (FLAG^+^, **B**), display of light chain (HA^+^, **C**), efficiencies of functional display (TNFα^+^ cells/HA^+^ cells, **D**) were presented as mean ± S.E. (*n*=3 independent experiments) with Student's *t*-test being performed, *P < 0.05. Effects of four ERSs (KDEL, HDEL, FEHDEL, and WEHDEL) were tested and compared to the clones without ERS.

We hypothesized that the ER retention sequence (ERS), a specific short sequence that mediates protein retention in the ER (Munro and Pelham, 1987; Mei et al., 2017), can extend the residence time of Fab fragments in yeast ER and thus facilitate their proper folding and assembly. Infliximab was our primary target because of its relatively low yeast surface display efficiency (Table S1). To determine the effect of ERS on Fab assembly, five ERSs of different strength were fused to the C-terminus of Infliximab light chain (V_L_-C_L_) (Figure 4A). Induced cells were labeled with anti-FLAGi-Fluor647 for detecting heavy chain display, anti-HA-FITC for detecting Fab assembly, and TNFα-His_6_ and anti-His_6_-iFluor647 for detecting Fab function. Similar to effects of tested molecular chaperons (Figure 3), fusion with ERSs decreased the surface display amounts of Infliximab heavy chain (FLAG^+^ cells) and assembled Fab (HA^+^ cells) (Figures 4B,C). However, the percentages and MFIs of TNFα^+^ cells increased as ERS strength increased (Figure 4D, Table S2). When 1 nM TNFα was used for labeling, TNFα^+^ % increased with the efficiency of functional Infliximab Fab display (TNFα^+^ cells / HA^+^ cells) improved from 29 ± 2% without ERS, to 42 ± 2% with weak ERS KDEL, and to 55 ± 4% and 64 ± 2% with strong ERSs FEHDEL and WEHDEL, which represented a 2.0- and 2.3-fold of improvement (Figure 4D). When labeled with 0.1 nM TNFα, strong ERSs FEHDEL and WEHDEL comparably enhanced functional display efficiency from 16 ± 1% to 33 ± 4% and 39 ± 2%, with increased percentages and MFIs of TNFα^+^ cells. Similar to Infliximab Fab, strong ERSs FEHDEL and WEHDEL increased percentages and MFIs of TNFα^+^ cells and prompted the functional display efficiency of D2E7 Fab for 1.5- and 1.9-fold (Figure S5, Table S3), while weak ERSs, HDEL, and KDEL, did not significantly affect display amounts or functional display efficiencies. Overall, these results suggested that ERSs with high retention strength improved the quality of yeast surface displayed Fab, presumably due to extended residence time in the ER that facilitated the formation of functional Fabs.

### Enrichment of a High Affinity Fab Clone From Spiked Libraries

**Figure 5:**
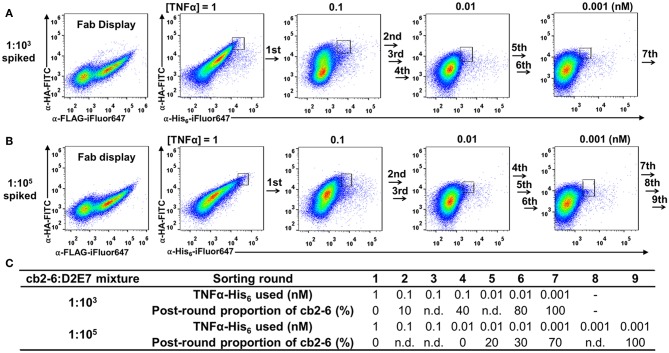
FACS enrichment for high affinity cb2-6 Fab clone from **(A)** 1:10^3^ and **(B)** 1:10^5^ spiked libraries. Cells carrying cb2-6 Fab gene were mixed with cells carrying D2E7 Fab gene at an initial ratio of 1:10^3^ or 1:10^5^. After induction, mixed cells were incubated with TNFα-His_6_ of decreasing concentration over the rounds and 0.1 μM anti-His_6_-iFluor647. Sorting gates with their population percentages of representative rounds are shown. After each round of sorting, 10 clones were randomly picked for plasmid extraction and DNA sequencing. **(C)** TNFα-His_6_ concentrations and post-round proportion of cb2-6 clones for each sorting rounds. n.d., not determined.

To validate the feasibility of yeast surface Fab display for affinity maturation, we mimicked the enrichment procedure with spiked libraries. Compared to D2E7 scFv of 0.96 nM binding potency, its variant cb2-6 scFv exhibited a reported affinity of 1.1 pM (Rajpal et al., 2005). When 10 pM TNFα was used for labeling yeast cells displaying D2E7 or cb2-6 Fab fragments, 9.6 or 20% cells were TNFα^+^, respectively (Figure 2C), suggesting the possibility to efficiently isolate cb2-6 from D2E7. Cells bearing cb2-6 Fab gene were mixed with cells bearing D2E7 Fab gene at a ratio 1:10^3^, and mixed cells were cultured for Fab expression. When labeled with anti-FLAG-iFluor647 and anti-HA-FITC, 43% of mixed cells were double positive indicating the successful display of assembled Fabs (Figure 5A, left panel). In the first round of FACS sorting (R1), 10^8^ mixed cells were labeled with 0.1 μM anti-HA-FITC for detecting Fab assembly, and 1 nM TNFα-His_6_ plus 0.1 μM anti-His_6_-iFluor647 for detecting TNFα binding. Top 1.0% double positive clones, equivalent to 10^6^ cells, were collected. Similar FACS sorting was performed for six more rounds with gradually reduced TNFα concentrations−100 pM for R2, R3, and R4; 10 pM for R5 and R6; and finally 1 pM for R7. For each round, 10^7^ cells were sorted, and 5 × 10^4^-7 × 10^4^ cells with the highest signals on both Fab display and TNFα binding were collected, equivalent to a selection gate of 0.60–0.72% (Figure 5A right panels). After certain rounds of FACS, 10 clones were randomly picked for plasmid extraction and DNA sequencing. Results indicated that the proportions of cb2-6 clones were enriched to 10% post-R2 and 40% post-R4, and reached to 80% post-R6 and 100% post-R7 (Figure 5C).

Successful isolation of cb2-6 from a 1:10^3^ mixture encouraged us to further test a more diluted library. The cb2-6 Fab cells was mixed with the D2E7 Fab cells at a ratio of 1:10^5^, a library size needed for affinity maturation practices (Boder and Wittrup, 1997). After induction for Fab expression and display, 43% of cells were FLAG^+^ and HA^+^ double positive (Figure 5B, left panel). Starting with this 1:10^5^ spiked library, total nine rounds of FACS were performed with decreasing TNFα concentrations from 1 nM to 1 pM (Figures 5B,C). 10^9^ mixed cells in R1 and 10^7^-10^8^ cells in later rounds were subjected for sorting with selection gates of 0.60–0.90% for the cells with the highest double signals on Fab display and TNFα binding. DNA sequencing results of randomly picked post-sorting clones indicated that cb2-6 increased its proportions to 20 and 40% post-R5 and post-R6, and achieved dominancy of 70% post-R7 and finally 100% post-R9.

## Discussion

Currently, scFv is the antibody format commonly used for yeast surface display (YSD) (Feldhaus and Siegel, 2004). Absent in natural IgGs, the introduction of an artificial flexible peptide linker, such as (G_4_S)_3_, between V_H_ and V_L_ domains can result in paratopic conformations different from these of intact IgGs (Gu et al., 2010). Although usually subtle and satisfactory for antigen binding specificity, such conformational variations can be troublesome for quantitative tasks such as affinity maturation (Yang et al., 2018). Preserving the entire antigen binding region, it has been suggested that Fab format retains the natural conformations and thus were widely used for determining the structures of antigen-antibody complex (Rothlisberger et al., 2005). Additionally, Fabs were more suitable for YSD of various antibody clones than scFvs (Sivelle et al., 2018). In this study, scFvs of D2E7 and its mutants A1, cb1-3, and cb-6 were also constructed for YSD (Figure S6A). Fluorescent staining for surface display and followed FACS analysis showed that scFv display levels varied from 31 to 46% (Figure S6B). In contrast, the same Ab clones in their Fab format showed a similar Fab display level with a narrow disparity of 41–43% (Figure 1B middle panels). Furthermore, for Infliximab, only its Fab but not scFv can be well-displayed on yeast cell surface (Sivelle et al., 2018). Collectively, our results, consistent with others (Rosowski et al., 2018; Wang et al., 2018; Yang et al., 2018), suggested that Fab is a reliable and practicable format for YSD.

As the largest organelle of most eukaryotic cells, the endoplasmic reticulum (ER) is the site where secretory polypeptides fold into their correct three-dimensional conformations, assemble into multi-subunit proteins, and achieve covalent modifications such as disulfide bond formation and initial glycosylation (Cooper and Hausman, 2000). By fusing ER targeting signals upstream of both heavy and light chains, Fabs successfully assembled and secreted for functional surface display (Figure 1). Interestingly, only around 40–45% of induced cells presented assembled Fabs on cell surface, which could be attributed to either inefficient induction/expression or improper domain folding/assembly. As folding and processing of polypeptide chains in the ER is facilitated by the molecular chaperones (Nishikawa et al., 2005; Buck et al., 2007), mounting evidence suggests that the secretion of scFvs increased with co-expression of a wide range of molecular chaperones (Shusta et al., 1998; Wentz and Shusta, 2007). In this study, a major member of the Hsp70 chaperone family Kar2p or the ER-associated Pdi1p was co-expressed to assist Fab production and surface display. Consistent with Kar2p functions, our results suggested that when assisted with Kar2p, the absolute amounts of Fab display (percentage and MFIs of HA^+^ cells) decreased, while functional display efficiency (defined as the ratio of cells displaying functional Fab over cells displaying assembled Fab) significantly improved for both D2E7 and Infliximab Fabs (Figure 3). Similarly, co-expression of Pdi1p also improved efficiency of functional display for Fabs in our study (Figure 3). Notably, the improvements were more significant for Infliximab (2.6- and 3.6-fold with Kar2p and Pdi1p) than D2E7 (1.5- and 2.0-fold, respectively). When inactive Kar2p or Pdi1p variants were co-expressed for comparison, Fab display amounts were decreased without improvements on functional display efficiencies (Figure S3, Table S1).

It was speculated that the co-expression of molecular chaperons might increase the burden of transcriptional and translational machinery in yeast cells. Indeed, qRT-PCR data suggested that the mRNA levels of co-expressed Kar2p or its variants were increased 9- to 16-fold compared to the level of physiological Kar2p in the D2E7 and Infliximab Fab producing cells (Figure S7A). Similarly, the mRNA levels of co-expressed Pdi1p and its variants were increased 9- to 17-fold over the background Pdi1p (Figure S7B). In our studies, we also attempted to increase the Fab folding and assembly efficiencies through retaining the light chain (V_L_-C_L_) longer in the yeast ER by anchoring the ERS at its C-terminus (Figure 4). Theoretically, the stronger ERS will increase the retention time and concentration of V_L_-C_L_ domain in the yeast ER, thus enhancing the Fab folding and assembly efficiencies. Consistently, our results suggested that fusion with ERSs improved the quality of displayed Fabs (Figure 4, Figure S4). However, it was also noticed that retaining the V_L_-C_L_ domain in the yeast ER decreased the display amounts of both heavy and light chains along with the strength of ERS.

In summary, this study characterized four complementary approaches for optimization of Fab yeast surface display—divergent promoter, ER signal peptide, molecular chaperones, and ER retention sequences. All these findings highlighted the importance to consider tradeoffs between quality and quantity of Fab yeast surface display for individual antibody clones. Encouraged by the success of Fab YSD and the feasibility of affinity maturation through FACS, current efforts have been focusing on surveying novel approaches, e.g., secretory organelle manipulation, to further improving antibody YSD. As Fab YSD and coupled FACS provide intact paratopic conformations and quantitative analysis at the monoclonal level, we expect this technology holds great potential for numerous applications in monoclonal antibody discovery and engineering.

## Data Availability Statement

All datasets generated for this study are included in the article/Supplementary Material.

## Author Contributions

MM, JL, SW, and KL performed experiments. MM, XG, and LY designed the experiments and wrote the manuscript. BI and GZ were involved in analysis and interpretation of experimental data. LY conceived the idea and supervised the whole research. All authors read and approved the final manuscript.

### Conflict of Interest

Two patent applications related to this study have been filed. The authors declare that the research was conducted in the absence of any commercial or financial relationships that could be construed as a potential conflict of interest.
