# Supplementary material for: Prompting Fab Yeast Surface Display Efficiency by ER Retention and Molecular Chaperon Co-expression
Source: Front Bioeng Biotechnol. 2019 Nov 26;7:362. doi: 10.3389/fbioe.2019.00362 (PMC6988814; doi:10.3389/fbioe.2019.00362)
Supplement: Supplementary file 1 [file Table_1.docx]

**Supplementary Materials**

**Prompting Fab Yeast Surface Display Efficiency by ER Retention and Molecular Chaperon Co-expression**

**Meng Mei^1^, Junhong Li^1^, Shengchen Wang^1^, Ki Baek Lee^2^, Brent L. Iverson^3^, Guimin Zhang^1^, Xin Ge^2^*, Li Yi^1^***

^1^State Key Laboratory of Biocatalysis and Enzyme Engineering, Hubei Collaborative Innovation Center for Green Transformation of Bio-resources, Hubei Key Laboratory of Industrial Biotechnology, School of Life Sciences, Hubei University, Wuhan, 430062, China

^2^Department of Chemical and Environmental Engineering, University of California, Riverside, CA, 92521, USA

^3^Department of Chemistry, University of Texas, Austin, TX 78712, USA

**Supplementary Tables**

**Table S1. Effects of Kar2p and Pdi1p on surface display of Fabs D2E7 and Infliximab**

|  | **FLAG^+^ cells** | | **HA^+^ cells** | | **TNFα^+^ cells** | | **Functional display efficiency**  **(TNFα^+^ % / HA^+^ %)** |
| --- | --- | --- | --- | --- | --- | --- | --- |
|  | **%** | **MFI** | **%** | **MFI** | **%** | **MFI** |  |
| **D2E7** | **43 ± 2** | **11473 ± 29** | **40 ± 2** | **58230 ± 81** | **14 ± 2** | **3018 ± 25** | **36 ± 2** |
| **D2E7 + Kar2p** | **25 ± 3** | **5619 ± 27** | **24 ± 2** | **3756 ± 27** | **13 ± 1** | **2664 ± 38** | **53 ± 1** |
| **D2E7 + Kar2p_E313A/K316A/S320A_** | **20 ± 2** | **4659 ± 41** | **19 ± 2** | **3190 ± 72** | **6.1 ± 1.1** | **1980 ± 23** | **32 ± 4** |
| **D2E7 + Kar2p_C63Y/F196L/G417S_** | **23 ± 2** | **4567 ± 52** | **21 ± 3** | **3213 ± 29** | **8.0 ± 2** | **2003 ± 17** | **38 ± 2** |
| **D2E7 + Pdi1p** | **23 ± 1** | **4986 ± 32** | **23 ± 2** | **3337 ± 61** | **16 ± 1** | **2682 ± 57** | **70 ± 1** |
| **D2E7 + Pdi1p_C4S_** | **25 ± 1** | **4912 ± 31** | **24 ± 1** | **3517 ± 76** | **7.4 ± 0.9** | **1992 ± 81** | **31 ± 1** |
| **D2E7 + Pdi1p_C6S_** | **26 ± 2** | **5061 ± 48** | **21 ± 4** | **3392 ± 81** | **7.8 ± 1.3** | **1945 ± 38** | **37 ± 2** |
| **D2E7 + Kar2p + Pdi1p** | **21 ± 2** | **4561 ± 46** | **20 ± 3** | **3007 ± 21** | **13 ± 1** | **2391 ± 19** | **65 ± 2** |
| **Infliximab** | **37 ± 3** | **5707 ± 81** | **33 ± 1** | **4724 ± 91** | **4.9 ± 1.3** | **2180 ± 19** | **15 ± 1** |
| **Infliximab + Kar2p** | **20 ± 2** | **4430 ± 100** | **23 ± 2** | **3315 ± 45** | **8.9 ± 2.1** | **2708 ± 72** | **39 ± 2** |
| **Infliximab + Kar2p_E313A/K316A/S320A_** | **21 ± 3** | **3607 ± 19** | **18 ± 3** | **3495 ± 31** | **3.4 ± 1.3** | **1673 ± 62** | **19 ± 2** |
| **Infliximab + Kar2p_C63Y/F196L/G417S_** | **17 ± 1** | **3417 ± 22** | **15 ± 2** | **3218 ± 49** | **2.4 ± 1.1** | **1399 ± 27** | **16 ± 3** |
| **Infliximab + Pdi1p** | **31 ± 2** | **3972 ± 67** | **21 ± 2** | **3330 ± 40** | **11 ± 2** | **2945 ± 59** | **53 ± 2** |
| **Infliximab + Pdi1p_C4S_** | **15 ± 2** | **3250 ± 38** | **16 ± 2** | **3392 ± 55** | **2.7 ± 1.2** | **1569 ± 32** | **17 ± 1** |
| **Infliximab + Pdi1p_C6S_** | **19 ± 1** | **3928 ± 57** | **18 ± 1** | **3617 ± 31** | **2.7 ± 0.8** | **1692 ± 19** | **15 ± 3** |
| **Infliximab + Kar2p + Pdi1p** | **18 ± 1** | **3529 ± 42** | **16 ± 2** | **3409 ± 39** | **9.0 ± 2** | **2761 ± 53** | **56 ± 3** |

**MFI, mean fluorescence intensity.**

**Table S2. Effects of ERS on surface display of Fab Infliximab**

|  | **FLAG^+^ cells** | | **HA^+^ cells** | | **TNFα^+^ cells**  **(with 0.1 nM TNFα)** | | **Functional display efficiency**  **(TNFα^+^ % / HA^+^ %)** | **TNFα^+^ cells**  **(with 1 nM TNFα)** | | **Functional display efficiency (TNFα^+^ % / HA^+^ %)** |
| --- | --- | --- | --- | --- | --- | --- | --- | --- | --- | --- |
|  | **%** | **MFI** | **%** | **MFI** | **%** | **MFI** |  | **%** | **MFI** |  |
| **Infliximab** | **35 ± 2** | **5707 ± 81** | **33 ± 1** | **4724 ± 91** | **5.1 ± 0.9** | **2179 ± 19** | **16 ± 1** | **9.4 ± 2.4** | **3009 ± 19** | **29 ± 2** |
| **+ KDEL** | **29 ± 1** | **3984 ± 39** | **27 ± 2** | **3158 ± 23** | **4.2 ± 0.8** | **1829 ± 53** | **15 ± 3** | **11 ± 2** | **3103 ± 26** | **42 ± 2** |
| **+ HDEL** | **26 ± 3** | **3701 ± 73** | **25 ± 1** | **3138 ± 38** | **5.9 ± 1.3** | **2287 ± 35** | **24 ± 1** | **12 ± 3** | **3226 ± 17** | **49 ± 2** |
| **+ FEHDEL** | **25 ± 2** | **3456 ± 61** | **22 ± 2** | **2820 ± 65** | **7.2 ± 2.5** | **2533 ± 51** | **33 ± 4** | **12 ± 3** | **3465 ± 56** | **55 ± 4** |
| **+ WEHDEL** | **23 ± 2** | **3394 ± 79** | **21 ± 3** | **2656 ± 98** | **8.1 ± 2.0** | **2686 ± 42** | **39 ± 2** | **13 ± 2** | **3622 ± 31** | **64 ± 2** |

**MFI, mean fluorescence intensity.**

**Table S3. Effects of ERS on surface display of Fab D2E7**

|  | **FLAG^+^ cells** | | **HA^+^ cells** | | **TNFα^+^ cells**  **(with 0.1 nM TNFα)** | | **Functional display efficiency (TNFα^+^ % / HA^+^ %)** |
| --- | --- | --- | --- | --- | --- | --- | --- |
|  | **%** | **MFI** | **%** | **MFI** | **%** | **MFI** |  |
| **D2E7** | **43 ± 2** | **11473 ± 29** | **40 ± 2** | **58230 ± 81** | **14 ± 2** | **3018 ± 25** | **36 ± 2** |
| **+ KDEL** | **40 ± 3** | **10330 ± 47** | **38 ± 4** | **5240 ± 100** | **13 ± 3** | **2840 ± 87** | **34 ± 3** |
| **+ HDEL** | **39 ± 1** | **9950 ± 16** | **38 ± 1** | **5250 ± 31** | **14 ± 2** | **2830 ± 26** | **37 ± 2** |
| **+ FEHDEL** | **33 ± 1** | **9700 ± 89** | **31 ± 3** | **4930 ± 53** | **17 ± 1** | **3660 ± 19** | **54 ± 1** |
| **+ WEHDEL** | **32 ± 3** | **8780 ± 43** | **28 ± 4** | **4240 ± 58** | **18 ± 3** | **3930 ± 39** | **65 ± 3** |

**MFI, mean fluorescence intensity.**

**Supplementary Figures**

**Figure S1**

**
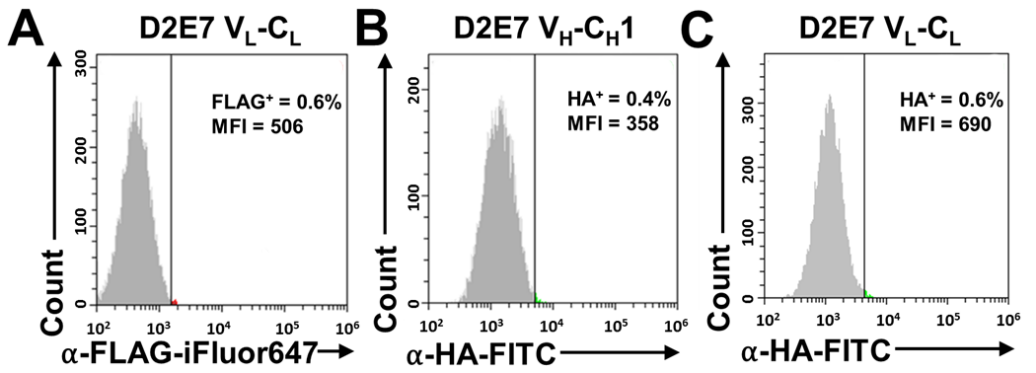
**

**Figure S1. Yeast surface display of Adalimumab D2E7 light chain or heavy chain alone. (A)** Flow cytometry analysis of cells carrying the light chain gene (V_L_-C_L_) of Adalimumab D2E7 without its heavy chain gene, after induction in SG-CAA medium and labeling with 0.1 μM anti-FLAG-iFluor647. **(B)** Flow cytometry analysis of cells carrying the heavy chain gene (V_H_-C_H_1) of Adalimumab D2E7 without its light chain gene, after induction in SG-CAA medium and labeling with 0.1 μM anti-HA-FITC. **(C)** Flow cytometry analysis of cells carrying the light chain gene (V_L_-C_L_) of Adalimumab D2E7 without its heavy chain gene, after induction in SG-CAA medium and labeling with 0.1 μM anti-HA-FITC. MFI, mean fluorescence intensity.

**Figure S2**

**
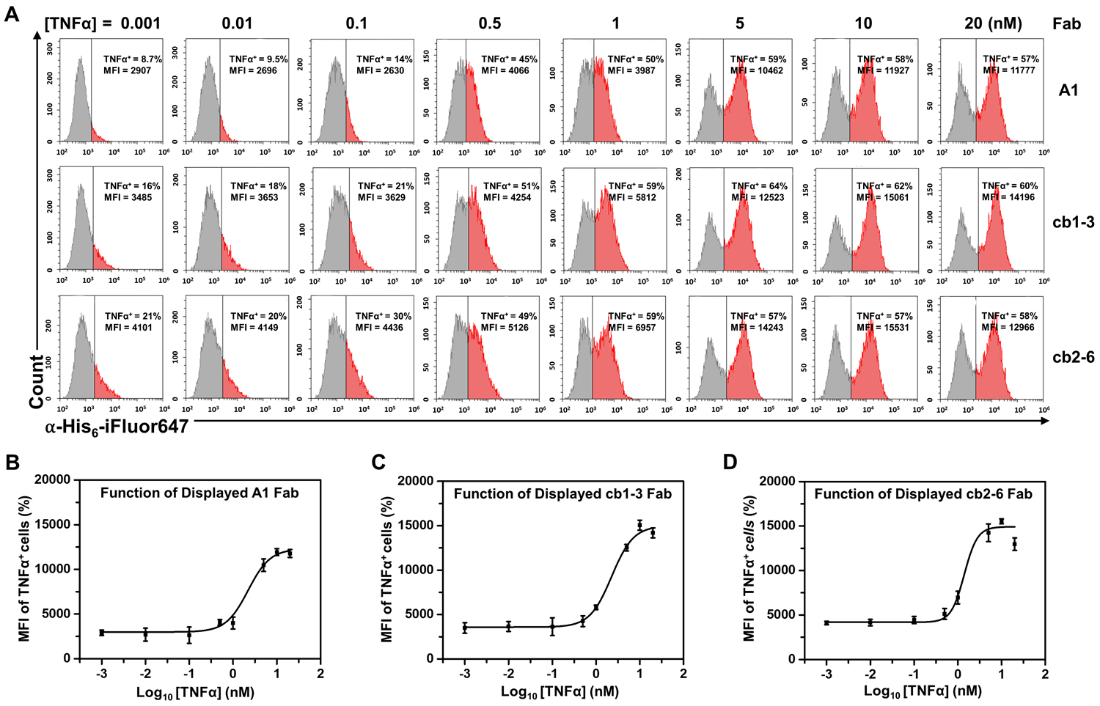
**

**Figure S2. Functional display of Fabs for Adalimumab variants A1, cb1-3, and cb2-6. (A)** Cells carrying Fab display constructs were induced with SG-CAA medium, and labeled with 0.001-20 nM TNFα-His_6_ and 0.1 μM anti-His_6_-iFluor647. Percentages and fluorescence intensities of TNFα^+^ cells were quantified by FACS. **(B-D)** Sigmoidal curves depicting fluorescence intensities of TNFα^+^ cells as a function of TNFα concentrations. Fab clones A1, cb1-3, and cb2-6 were tested. Data are presented as mean ± S.E. (n=3 independent experiments). MFI, mean fluorescence intensity.

**Figure S3**


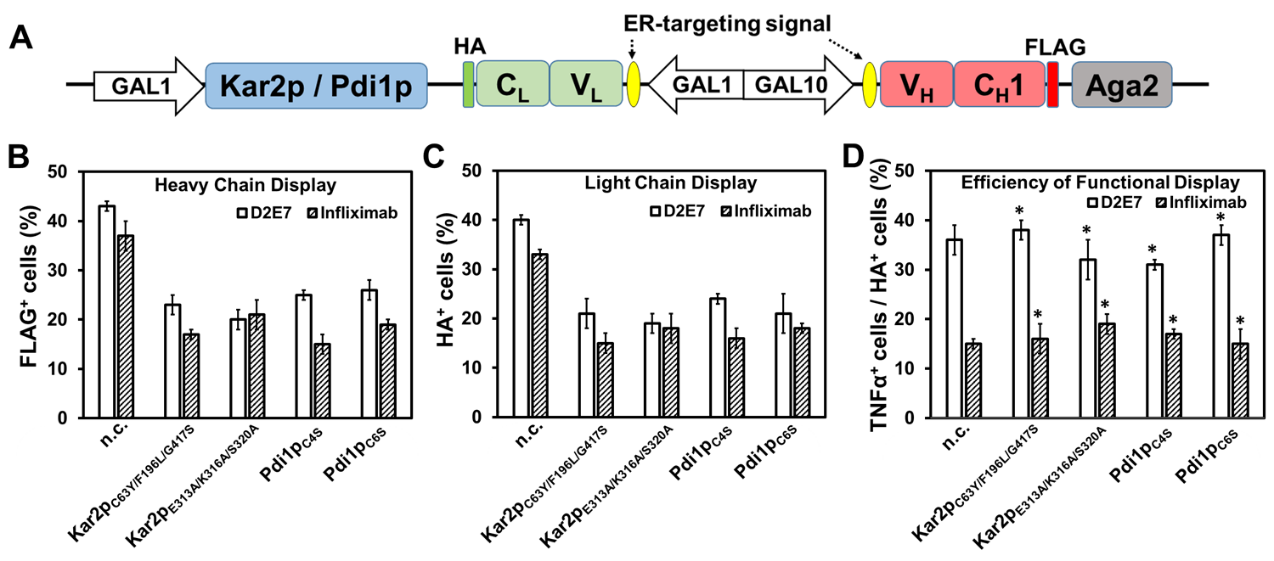


**Figure S3. Effects of co-expression of Kar2p or Pdi1p variants on Fab functional display of D2E7 and Inflixmab. (A)** Co-expression of Kar2p or Pdi1p variant was under the control of *GAL1* promoter on pESD-Fab-Pdi1p/Kar2p variant. 0.1 μM anti-FLAG-iFluor647, 0.1 μM anti-HA-FITC, and 0.1 nM TNFα / 0.1 μM anti-His6-iFluor647 were used for labeling induced cells. Display of heavy chain (FLAG+, panel **B**), display of light chain (HA+, panel **C**), efficiencies of functional display (defined as the percentage ratios between TNFα+ cells and HA+ cells, panel **D**) were presented as mean ± S.E. (n=3 independent experiments) with Student’s t-test being performed, *P<0.05.

**Figure S4**

**
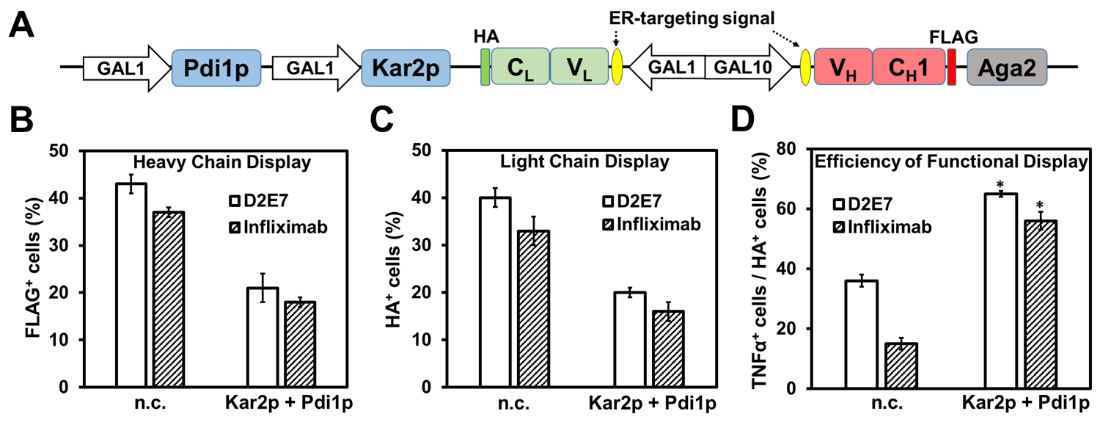
**

**Figure S4. Effects of simultaneous co-expression of both Kar2p and Pdi1p on Fab functional display of D2E7 and Inflixmab. (A)** Simultaneous co-expression of Kar2p or Pdi1p was under the control of additional *GAL1* promoters on pESD-Fab-Pdi1p-Kar2p. 0.1 μM anti-FLAG-iFluor647, 0.1 μM anti-HA-FITC, and 0.1 nM TNFα / 0.1 μM anti-His_6_-iFluor647 were used for labeling induced cells. Display of heavy chain (FLAG^+^, panel **B**), display of light chain (HA^+^, panel **C**), efficiencies of functional display (defined as the percentage ratios between TNFα^+^ cells and HA^+^ cells, panel **D**) were presented as mean ± S.E. (n=3 independent experiments) with Student’s t-test being performed, *P<0.05.

**Figure S5**

**

**

**Figure S5. Effects of ER-retention sequence (ERS) on Fab D2E7 functional display.** ERSs were fused to the C-terminus of D2E7 light chain. 0.1 μM anti-FLAG-iFluor647, 0.1 μM anti-HA-FITC, and 0.1 nM TNFα / 0.1 μM anti-His_6_-iFluor647 were used for labeling induced cells. Display of heavy chain (FLAG^+^, panel **B**), display of light chain (HA^+^, panel **C**), efficiencies of functional display (TNFα^+^ cells / HA^+^ cells, panel **D**) were presented as mean ± S.E. (n=3 independent experiments) with Student’s t-test being performed, *P<0.05. Effects of four ERSs (KDEL, HDEL, FEHDEL, and WEHDEL) were tested and compared to the clones without ERS.

**Figure S6**

**
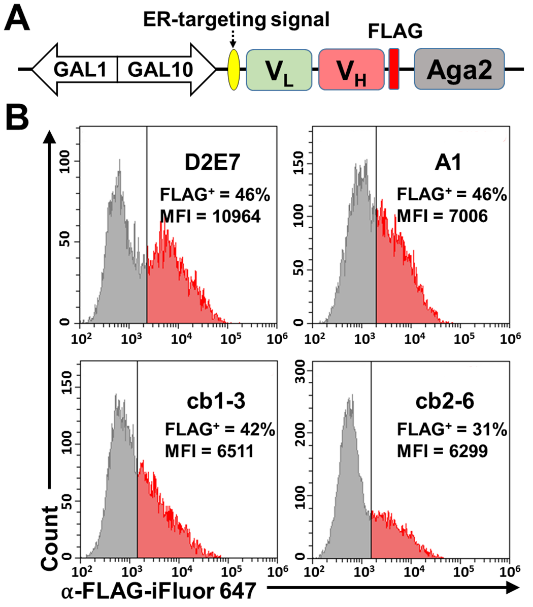
**

**Figure S6. Yeast surface display of Adalimumab scFv variants. (A)** pESD-scFv. Expression cassettes for V_L_-V_H_ was located at the downstream of GAL10 promoter and ER-targeting signals. A (G_4_S)_3_ linker was inserted between V_L_ and V_H_, and the scFv construct was fused with a FLAG tag to the N-terminus of Aga2. **(B)** Validation of scFv display on yeast cell surface by FACS. Expression was induced with SG-CAA medium and cells were incubated with 0.1 μM anti-FLAG-iFluor647. Four scFvs of Adalimumab variants were tested. Display efficiencies, defined as percentages of cells presenting scFv (FLAG^+^ cells), was shown in boxes. MFI. mean fluorescence intensity.

**Figure S7**


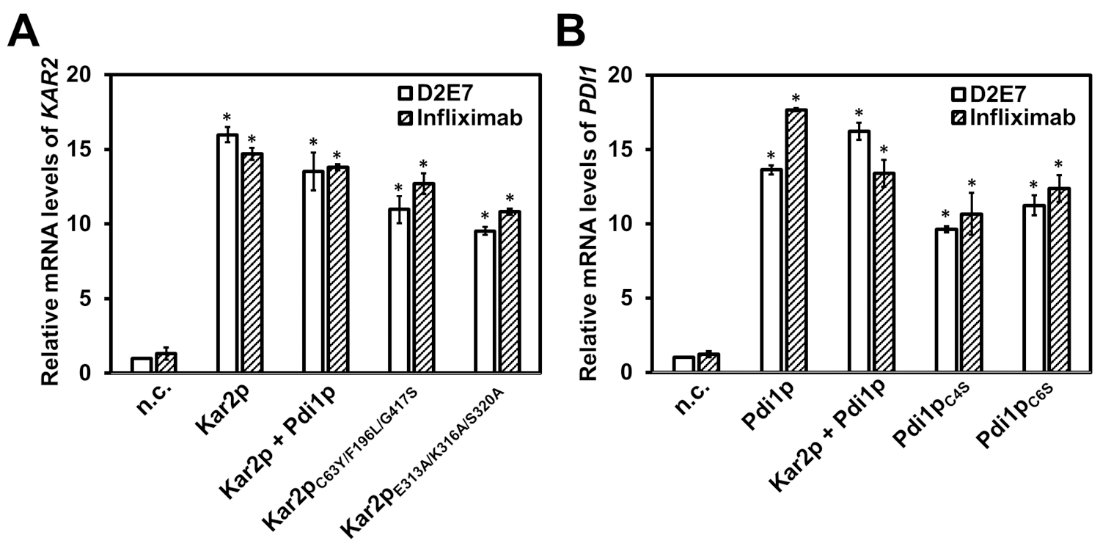


**Figure S7. Relative mRNA levels of *KAR2* and *PDI1* in cells co-expressing Kar2p, Pdi1p, or their variants. (A)** Relative mRNA levels of *KAR2* in D2E7, Infliximab, D2E7-Kar2p, Infliximab-Kar2p, D2E7-Kar2p-Pdi1p, Infliximab-Kar2p-Pdi1p, D2E7-Kar2p variants, and Infliximab-Kar2p variants cells. (**B**) Relative mRNA levels of *PDI1* in D2E7, Infliximab, D2E7-Pdi1p, Infliximab-Pdi1p, D2E7-Kar2p-Pdi1p, Infliximab-Kar2p-Pdi1p, D2E7-Pdi1p variants, and Infliximab-Pdi1p variants cells. The relative mRNA levels of *KAR2* and *PDI1* were normalized by themselves in EBY100 cells, and presented as mean ± S.E. (n=3 independent experiments) with Student’s t-test being performed, *P<0.05.
